# Older adults fail to form stable task representations during model-based reversal inference

**DOI:** 10.1016/j.neurobiolaging.2018.10.009

**Published:** 2019-02

**Authors:** Dorothea Hämmerer, Philipp Schwartenbeck, Maria Gallagher, Thomas Henry Benedict FitzGerald, Emrah Düzel, Raymond Joseph Dolan

**Affiliations:** a Institute of Cognitive Neuroscience, University College London, London, UK; b Institute of Cognitive Neurology and Dementia Research, Otto-von-Guericke University Magdeburg, Magdeburg, Germany; c German Centre for Neurodegenerative Diseases, Magdeburg, Germany; d Centre for Cognitive Neuroscience, University of Salzburg, Salzburg, Austria; e Neuroscience Institute, Christian-Doppler-Klinik, Paracelsus Medical University Salzburg, Salzburg, Austria; f The Wellcome Trust Centre for Neuroimaging, University College London, London, UK; g Oxford Centre for Functional MRI of the Brain, Nuffield Department of Clinical Neurosciences, University of Oxford, Oxford, UK; h Department of Psychology, Royal Holloway University of London, Egham, UK; i School of Psychology, University of East Anglia, Norwich, UK; j Max Planck–UCL Centre for Computational Psychiatry and Ageing Research, London, UK

**Keywords:** Decision-making, Task representations, Pupillometry, Model-based inference, Aging, Feedback evaluation

## Abstract

Older adults struggle in dealing with changeable and uncertain environments across several cognitive domains. This has been attributed to difficulties in forming adequate task representations that help navigate uncertain environments. Here, we investigate how, in older adults, inadequate task representations impact on model-based reversal learning. We combined computational modeling and pupillometry during a novel model-based reversal learning task, which allowed us to isolate the relevance of task representations at feedback evaluation. We find that older adults overestimate the changeability of task states and consequently are less able to converge on unequivocal task representations through learning. Pupillometric measures and behavioral data show that these unreliable task representations in older adults manifest as a reduced ability to focus on feedback that is relevant for updating task representations, and as a reduced metacognitive awareness in the accuracy of their actions. Instead, the data suggested older adults' choice behavior was more consistent with a guidance by uninformative feedback properties such as outcome valence. Our study highlights that an inability to form adequate task representations may be a crucial factor underlying older adults' impaired model-based inference.

## Highlights

• Older adults overestimate the changeability of task states in uncertain environments.
• Unreliable task representations impact model updating in older adults.
• Older adults focus on outcome valence rather than model updating during feedback.
• Older adults show reduced metacognitive awareness of their decision-making accuracy.
• Pupil diameter encodes model updating and not surprise in young and older adults.

## 1 Introduction

In situations of unreliable or changeable action-outcome relationships, we need to rely on abstract task representations to estimate the likely outcomes of our actions and make adaptive adjustments based on these outcomes. Evidence across several cognitive domains shows that older adults have difficulties in forming adequate task representations (Hämmerer et al., 2014). For instance, in conflict tasks, attentional filtering of inputs based on task representations is less pronounced or absent in older adults (Gazzaley, 2011, Hämmerer et al., 2010, Störmer et al., 2013). In decision-making, recent modeling studies show that older adults have difficulties in capturing uncertainties in task representations (Nassar et al., 2016) and show a weaker tendency in deploying model-based decision-making (Eppinger et al., 2013a, Eppinger et al., 2013b).

**Fig. 1:**
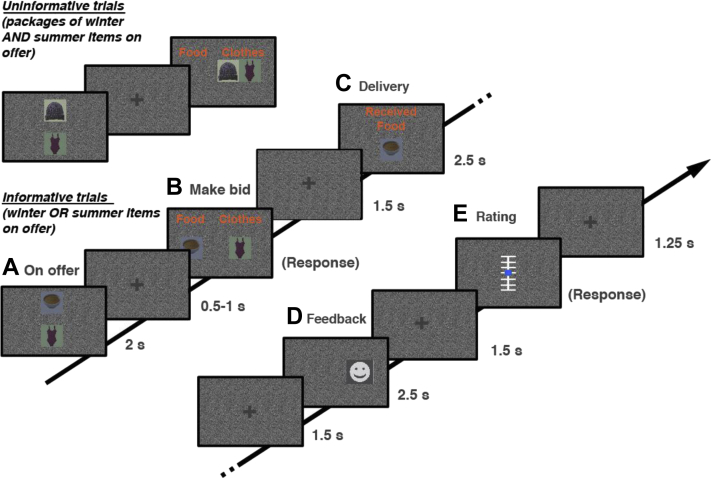
Probabilistic reversal task. Participants were asked to choose between a winter and summer item (A) according to their belief that the current season was either summer or winter. Correct seasonal choices (and correct deliveries) resulted in 87% of the trials in a profitable or unprofitable sale [happy or sad face in (D), respectively]. To balance gains and losses, the delivery service (C) would not always bring the desired item. Moreover, half of the trials were uninformative for learning about the current season (i.e., did not allow a choice between winter or summer items). After every trial, participants were asked to rate how sure they were that the current season was summer or winter (E). Background, font color, as well as pictures where adjusted to an overall luminance of 0.5 to allow for continuous pupillometric recordings. (For interpretation of the references to color in this figure legend, the reader is referred to the Web version of this article.)

Here we investigate the question of formation of task representations and their influence on decision-making in older adults, by comparing choice performance of young and older adults in a model-based reversal learning task (see Fig. 1). The task required subjects to infer their current state, framed as to whether the current season is winter or summer, and to also detect switches between the seasons. Participants were not explicitly informed of what the current season was, but inferred this from sales of winter-specific or summer-specific items. In simple terms, the season was a hidden state. Switches between task states (winter or summer) occurred with a certain frequency (reversal probability) and feedback for correct responses (choosing the seasonally appropriate item) was reliable in 87% of choices (outcome predictability). Participants were informed about the underlying uncertainty in outcome prediction, but were not told about the precise reversal probability or outcome predictability.

To examine behavior in a fine-grained manner, we used computational modeling which assessed how these 2 types of uncertainty were expressed in subjects' choices. Based on prior evidence suggestive of inaccurate task representations in older adults (Nassar et al., 2016), we predicted older adults would be impaired in representing task uncertainties. Furthermore, as we wanted to investigate whether an inability to form precise task representations impacts on the evaluation of action outcomes, we recorded changes in pupil diameter during outcome feedback. Pupil diameter is a proxy measure for increased activity of the noradrenergic system, as pupils dilate when increased neuronal activity is triggered in the locus coeruleus (LC) (Joshi et al., 2016). Current theories regarding the role of noradrenergic modulation in higher cognitive functions suggests increased noradrenergic modulation during events that are (subjectively) relevant in a given task context (Aston-Jones and Cohen, 2005, Dayan, 2012, Sara and Bouret, 2012, Yu and Dayan, 2005). For instance, a common finding is increased phasic firing to target stimuli in an oddball task (Aston-Jones and Cohen, 2005). Interestingly, Aston-Jones et al showed that nontarget stimuli in oddball tasks are not encoded in phasic LC firing, even if they are as rare and as unexpected in occurrence as target stimuli (Aston-Jones et al., 1994). Furthermore, activity in the noradrenergic LC is seen for both aversive and appetitive stimuli, as long as these are important in a given task context (e.g., novel stimuli, stimuli indicating the necessity to alter behavior) (Berridge and Waterhouse, 2003). This supports the idea that the noradrenergic system indexes the (subjective) salience of events given their relevance in a particular task context (Berridge and Waterhouse, 2003).

We used pupil diameter recordings to infer the subjective salience of feedback for events that either did, or did not, allow updating of current task representations. We expected that feedback that allowed updating of task representations would be more subjectively salient and therefore lead to an increased pupil diameter. By contrast, inadequate task representations would be expected to be reflected in a reduced ability to differentiate between feedback that is relevant or irrelevant in updating task representations. To stringently test for the relevance of task representations during feedback evaluation we included 2 experimental manipulations.

First, half of the trials were uninformative for learning about the current season, as on these trials, there were sales of winter as well as summer items which disallowed an inference as to whether a successful sale was related to either winter or summer. Consequently, positive or negative outcomes on these trials were not informative for updating beliefs about the current season (task representation). This allowed us to decorrelate belief update from the pure unexpectedness of a stimulus (O’Reilly et al., 2013, Schwartenbeck et al., 2015). If subjects can successfully dissociate between these 2, then informative (seasonally specific) feedback should elicit a stronger pupillometric response as compared with uninformative (seasonally unspecific) feedback.

Second, above chance performance results in a clustering of negative outcomes around reversal time points in reversal learning tasks. This means that the information content imparted by a stimulus and its valence are often correlated in reversal learning tasks, rendering it problematic for assessing the relevance of task representations via individual responses to feedback as the salience of feedback will depend on its valence as well as its expectedness given a task representation. Unlike previous paradigms, our task design therefore orthogonalized information content (seasonal or nonseasonal items), expectedness (valid or invalid feedback) and valence of action outcomes. Specifically, choice accuracy was decoupled from outcome valence by introducing an unreliable delivery of chosen items (see Fig. 1). This way, the amount of gains and losses for (un-)expected outcomes could be balanced. Gain and loss outcomes were balanced for informative (seasonally specific) as well as uninformative (seasonally unspecific) trials. Unlike prior studies, this allowed us to investigate age differences in feedback evaluation based on task representations independent of age differences in the sensitivity to loss or gain outcomes or age differences in processing the expectedness of feedback (Hämmerer and Eppinger, 2012, Hämmerer et al., 2011, Larkin et al., 2007).

Finally, by orthogonalizing informativeness and expectedness of outcomes, we could address whether the mere unexpectedness of an event (surprise) can be differentiated from its informativeness for learning about task representations (model updating). There is currently an open debate whether the noradrenergic system is more responsive to events that are surprising given a particular task context (unexpected events that do not necessarily allow for learning about task states) or is only responsive to events that allow for model updating (unexpected events that allow for learning about task states) (Nassar et al., 2012, O’Reilly et al., 2013, Schwartenbeck et al., 2015). Previous studies report contradictory findings as to whether surprising events that allow for model updating are more salient, as evident in increased pupillometric responses during model-based learning (Nassar et al., 2012, O’Reilly et al., 2013). Here, we can examine age differences in the encoding of surprise and model updating in model-based learning. Surprise (how unexpected is an event given the task representation in case of uninformative feedback?) was formalized as the negative log probability of the event occurring, which we refer to as “Shannon surprise” (Schwartenbeck et al., 2013). Model updating (how much is the task representation changed in case of informative feedback?) was defined via the Kullblack-Leibler divergence from before posterior beliefs about task contingencies [often called “Bayesian surprise” (Itti and Baldi, 2006)].

## 2 Methods

### 2.1 Participants

A total of 25 healthy younger adults (15 female, mean age = 23.83) and 22 healthy older adults (11 female, mean age = 68.15) participated in the study. Data from 3 older adults had to be excluded because of difficulties in understanding task instructions (switching between task states in around 50% of the trials, N = 2) and faulty pupillometric recordings (N = 1), resulting in a final sample size of 25 younger adults and 19 older adults. Eligibility criteria were being aged between 20 and 30 years for younger adults and over 60 years for older adults, with normal or corrected to normal vision, English as a first language, and no history of psychiatric disorder. Younger adults were recruited from the UCL ICN subject database, whereas older adults were recruited via advertisements and contacted by phone and email to assess their eligibility. Written informed consent was obtained from the participants before starting the task, and the experiment was approved by the UCL ethics committee. Participants were paid £20 for taking part in the 2h study.

### 2.2 Experimental procedure

Task details are outlined in the Results and Fig. 1. The paradigm measured participants' ability to establish a model of the task contingencies and use this model to perform inference on a latent state in this task, operationalized as the current season (winter or summer, cf. Fig. 1). Specifically, participants were tasked to bid on either winter or summer items (e.g., warm clothes or bathing suits, hot soup or ice cream) depending on their beliefs about the current season. Participants were not informed about the current season but had to perform trial-by-trial inference on the season based on feedback in the task (operationalized as which seasonal items were currently selling better, see Fig. 1 for an illustration). Participants were informed that seasons would remain constant for an extended period of time and encouraged to detect switches in seasons as soon as possible. Importantly, half of the trials in this paradigm were informative whereas the other half of the trials were uninformative for learning about the current season. In informative trials, subjects could choose between a winter and a summer item and receive positive or negative feedback on their chosen item. Thus, in these trials, subjects could perform inference on the current season based on their chosen item and received feedback. In uninformative trials, subjects were forced to choose a “package” consisting of an unknown ratio of winter and summer items. Thus, in these trials, no inference about the current season could be made. The feedback in both conditions was probabilistic and had a predictability or validity of 87% (i.e., 87% chance of receiving a gain for selling the correct seasonal item on informative trials and the offered package on uninformative trials). In the computational modeling, we treated the estimated outcome predictability (as well as the estimated reversal probability of switches between seasons) as a free parameter that was estimated based on observed behavior (see the following).

Finally, on informative as well as on uninformative trials, an unreliable delivery service was introduced to decorrelate choice accuracy from feedback valence. Importantly, this task design allowed us to disentangle the effects for positive and negative, informative and uninformative, as well as expected and unexpected feedback in probabilistic reversal learning. To allow for a clear separation of reversals and probabilistic feedback, trials with invalid feedback were predefined and did not occur within 2 trials of task state reversals. At the end of each trial, participants were asked to indicate what the current task state (season) is as well as how certain they were in their assessment on a 7-point Likert scale (cf. Fig. 1).

The task consisted of 324 trials in total separated into 4 blocks of 81 trials each. As part of the remuneration, a bonus payment of £6 was available, depending on the rating accuracy (thresholded at 70% per block) as well as the gains in the task (thresholded at 50 points per block, with gains counting as 2 points and losses counting as minus 1 point). Once the task had been completed, participants were given a questionnaire about their current mood, their opinions on the task's difficulty, their motivation to do well in the task, and whether they had used any particular strategies for choosing the correct seasonal item. Participants completed an extensive practice session consisting of 50 trials before starting the task to ensure that all participants understood the task.

### 2.3 Materials

The experimental task was programmed using MATLAB R2015a (version 8.5.0.197613, the MathWorks, Inc, 2015) and Cogent 2000 software. Stimuli were presented on a computer screen and viewed from a distance of 80 cm. All images were controlled to a luminance of 50%. Head position was stabilized using a chin and headrest. Participants' pupil measurements were recorded using EyeLink 2000 eye-tracker (SR Research, 35 Beaufort Dr, Kanata, ON K2L 2B9, Kanada). Eye-tracking and pupil data were analyzed in MATLAB and FieldTrip (version 20140615, Oostenveld, Fries, Maris, and Schoffelen, 2011) software. Participants' choices of task stimuli and certainty ratings were recorded using 2 four-choice button boxes. Data were analyzed in SPSS version 21 (IBM Corp., 2012).

### 2.4 Acquisition and preprocessing of pupillometric data

Pupillometric data were recorded from the right eye with a sampling rate of 1000 Hz. Pupillometric data were collected continuously during the task. Eye-tracking calibration was conducted before the start of each task block. For preprocessing, data were segmented 500 ms before and 2500 ms after feedback onset and 1750 ms before and 500 ms after cue onset. A custom-made MATLAB script detected eye-blinks in segmented data based on pupil diameter. Periods of missing data because of blink-related eye artifacts were cut out in time windows of 200 ms and 30 ms around large and small artifacts, respectively, and replaced by linear interpolation. Trials were then individually inspected and excluded if they contained excessively noisy or missing data (on average, 10.8% of the trials were removed in younger adults and 10.1% in older adults). For the assessment of phasic pupil diameters, pupil data were baseline-corrected with respect to a window 200 ms before outcome onset, and z-scored per individual to allow comparison of phasic pupil diameter changes across trial types independent of interindividual differences in pupil diameter changes across the task.

### 2.5 Analysis of pupillometric data

Cleaned pupillometric data were analyzed with respect to condition and age differences in changes of pupil diameter across the task. We used general linear model (GLM) analyses to examine whether trialwise changes in model updating or surprise were reflected in pupil dilation. Specifically, in a first-level analysis, trialwise regressors of interest were entered in 1 GLM per person to predict trial-by-trial changes in pupil diameter with different amounts of model updating as well as surprise. In addition, regressors indicating gain versus loss trials, and trials where responses between seasons were changed [as increased LC firing was observed for response reversals in animals and increased pupil diameters might thus just indicate the decision to switch (Bouret and Sara, 2004)]. This allowed us to assess changes in pupil diameter with model updating and surprise in the course of learning independent of more noticeable trial properties or categorical differences between trials which might result in independent effects on pupil diameters. To assess effects of model parameters on pupil diameters independent of interindividual differences in model parameters, individual regressors were z-scored. Significant differences in pupil diameter between conditions as well as between age groups were assessed with permutation tests (compared against time series with randomly shuffled condition labels across trials within participants [100 repetitions] or age group labels [1000 repetitions], respectively). Permutation analyses were chosen because pupil diameter measures can be assumed to be autocorrelated across sample points. In this manner, we provided comparison against intact sequences of pupil diameter changes, which had the same extent of autocorrelation. A further advantage of GLM analyses on pupil data is that condition effects as well as age differences in condition effects can be assessed independently of age differences in mean pupil diameter changes across conditions (which are captured in the intercept of the regression analyses).

Younger and older adults pupil data did not differ in the number of excluded trials because of artifacts or noisy recordings (*t*(1,42) = −0.7, *p* = 0.86) as well as baseline noise in pupil diameter recordings (*t*(1,42) = −1.6, *p* = 0.12; assessed as mean of standard deviation in time window 200 ms before feedback as well as onset of fixation cross before cue presentation). We would thus not assume to see age differences in pupil data solely because of differences in measurement properties.

### 2.6 Analyses of behavioral and model data

**Fig. 2:**
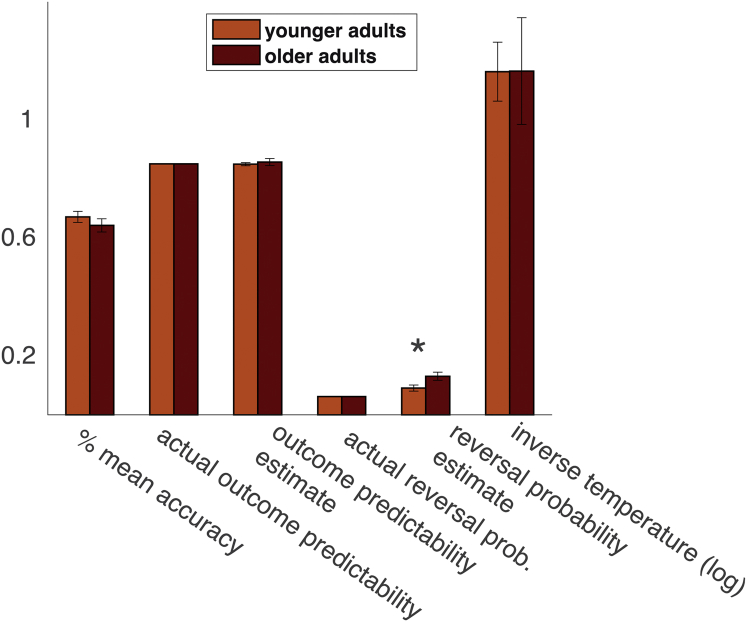
Age differences in behavioral performance and model estimates of Bayesian inference on probabilistic reversal learning task. Age groups did not differ in overall accuracy of choices or overall estimates of the outcome predictability, which were close to the actual outcome predictability. However, older adults overestimate the frequency of task state reversals. Bars indicate means per age groups, error bars SEs, asterisks reliable group differences at *p* <0.05.

Individual behavioral data were analyzed as aggregated data across the whole task (mean choice accuracy, frequency of switching after gains, and losses, see Fig. 2) as well as aggregated across trials immediately after reversals (first 6 trials after reversals) and trials later on in learning (7–12 trials after reversals). Reaction times exceeding 3.5 SD as compared with median reaction times were excluded for each individual. Similarly, parameter estimates characterizing choice behavior across the whole task (estimated reversal probability, estimated reliability of feedback) were compared across age groups as well as aggregated across trials (belief about the season, belief updating) analogous to the behavioral data (first 6 trial after reversals vs. 7–12 trials after reversals) to assess learning after reversals. Nonparametric tests were used when data or model estimates were not normally distributed, greenhouse-geisser corrected results were reported when assumptions of sphericity were violated and t-tests for groups with unequal variances were reported when variances differed between age groups.

**Fig. 3:**
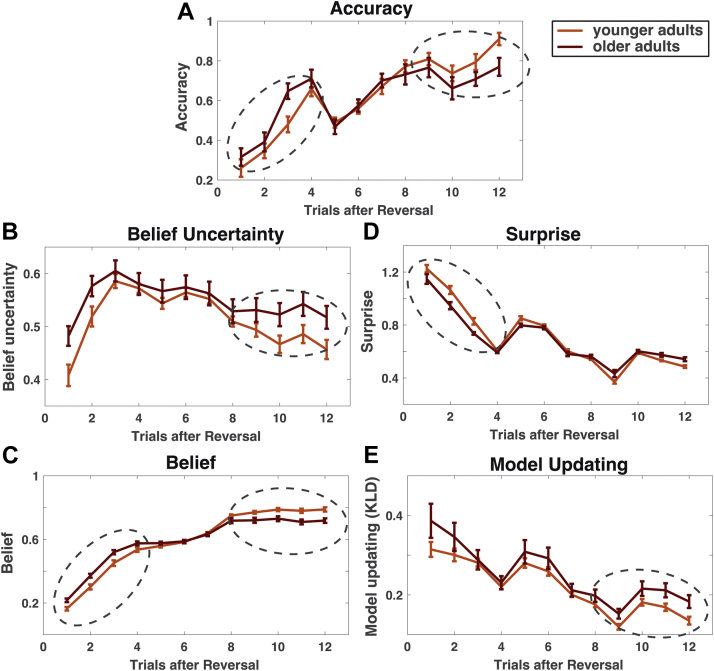
Age differences in trialwise accuracy and model estimates after reversals. [(A) Dip in accuracy around trial 5 is because of increased numbers of unexpected (probabilistic) feedback around trial 5 after reversals, see Methods]. Older adults show lower levels of correct choices (A), increased belief uncertainty (B) and lower belief estimates of the currently correct task state (C), especially in the second half of trials after reversals. This suggests that they struggle with converging on an unequivocal belief about the current season, even when beliefs about the current season should be established by consistent feedback. During feedback processing, surprise (D) is lower in older adults early after reversals whereas model updating (E) is higher in older adults later on after reversals. This suggests that older adults change beliefs more frequently than younger adults later on after reversals. Note that accuracy, surprise, and model updating are only reported for informative trials (cf. Fig. 1, choices on uninformative trials were defined to be correct if carried out as instructed and model updating was not possible for uninformative trials). Beliefs and belief uncertainty are shown for all trials as they reflect certainty and beliefs at the beginning of a trial before the informativeness of the trial was determined. Error bars indicate SEs. Dashed circles indicate reliable group differences in first or second half of trials after reversals.

Belief about the season was defined as the task state beliefs of the currently correct task state (beliefs independent of task state summer or winter, calculated as | *P*(task state = winter)−0.5 |) and generally increased after reversals (cf. Fig. 3B). The extent of belief or model updating (Fig. 3E) was determined by calculating the Kullblack-Leibler divergence (D_KL_) on the change from before posterior beliefs after making an observation at a given trial:

$$D_{KL}\left[P\left(x_{t}|o_{t},y_{t},x_{t-1},A,B\right)|P\left(x_{t}|x_{t-1},B\right)\right]=\sum_{i}P\left(x_{i,t}|o_{t},y_{t},x_{t-1},A,B\right)\cdot \ln\left(\frac{P\left(x_{i,t}|o_{t},y_{t},x_{t-1},A,B\right)}{P\left(x_{i,t}|x_{t-1},B\right)}\right)$$

Furthermore, we derived the trial-by-trial information-theoretic surprise (Fig. 3D) as a measure of the pure unexpectedness of an observation (O’Reilly et al., 2013, Schwartenbeck et al., 2016):

$$surprise=-\ln\sum_{i}P\left(o_{t}|x_{i,t},y_{t},A\right)\cdot P\left(x_{i,t}|x_{t-1},B\right)=-\ln\;P\left(o_{t}\right)$$

and belief entropy (Fig. 3C) as a measure of absolute uncertainty during decisions as follows:

$$H_{season}=\sum_{i}P\left(x_{i,t}|x_{t-1},B\right)\text{log}\left(P\left(\theta x_{i,t}|x_{t-1},B\right)\right)=E\left[\log\;P\left(x_{i,t}|x_{t-1},B\right)\right]$$

## 3 Results

We used a probabilistic reversal learning task (Fig. 1) to examine adult age differences in relying on adequate task representations in model-based reversal learning. As in a typical probabilistic reversal learning task, participants had to identify alternating task states (winter or summer—alternating with a certain reversal probability per trial [6%]) based on probabilistic feedback (successful sale of winter or summer items—with success of selling seasonally suitable items dependent on a certain preset outcome predictability [87%]).

On each trial (cf. Fig. 1), participants were first presented with the items available for bidding by the wholesaler (A). After making their choice (B), the received item was presented. Participants did not always receive the item that they bid on (C) (and this was attributed to an unreliable delivery service), to balance learning from losses and gains (i.e., balanced number of expected or unexpected gains and losses across trials, cf. supplementary Table 2). After being presented with the delivered item (C), participants were shown whether they made a gain from the sale of the delivered items (D). Outcomes were gains (smiling faces) or losses (frowning faces). Outcomes for correct seasonal items resulted in a profitable sale on about 87% of trials, with the 13% of losses attributed to unreliability in customer choices.

As outlined previously, feedback was uninformative for learning about seasons on half of the trials (participants had to bid on packages of mixed seasonal items, Fig. 1). Participants were explicitly instructed about the uninformativeness of these trials, that is, they were told that the ratio of summer or winter items within the package was unknown, and that gains or losses were thus not informative for learning about the current season. Participants were instructed to pick the offered package and informed that choosing the offered package would result in most of the cases in a profit. However, as in informative trials, offered packages were not guaranteed to result in a profit (due to probabilistic feedback), resulting in higher surprise (cf. supplementary Fig. 2). In addition, as in the case of informative trials, gains and losses were balanced for expected and unexpected outcomes under cover of an unreliable delivery service. This allowed us to isolate the relevance of task representations for feedback evaluation as well as to distinguish surprise from model updating.

### 3.1 Model comparison favors Bayesian inference models over learning models for probabilistic reversal learning

To estimate task representations underlying choice behavior, we compared a Bayesian inference (Hidden Markov) model, 3 Rescorla Wagner (RW) learning models, and a simple switching model (see Methods for details). Bayesian inference modeling derives trialwise regressors reflecting the strength of belief of being in a “Summer” or “Winter” state. At any given trial, a posterior belief is obtained using Bayes rule based on the prior belief about the current task state, the overall assumed predictability of task states as well as the current outcome history. Posterior beliefs are updated at each trial, taking into account also an assumed overall reversal probability of task states. Updated posterior beliefs served as a prior belief at the subsequent trial. The RW-learning models were (1) a standard RW-learning model in which each action value was updated independently for the 2 seasons, (2) a model in which values across seasons were simultaneously updated, (3) a model with an adaptive learning rate based on the size of prediction errors (see supplementary Table 1). Moreover, we tested a simple switching model where the expected value of a seasonal stimulus on the next trial was only determined by the feedback on the previous trial and not by beliefs or values of the current task state.

In keeping with previous findings on reversal learning tasks (Costa et al., 2015, in preparation; Hampton et al., 2006, Schlagenhauf et al., 2014, Wilson and Niv, 2012), model comparison favored the Bayesian inference model in both age groups (see supplementary Table 1). The winning model fitted 3 free parameters for every individual: an estimate of the reversal probability, an estimate of the outcome predictability and an inverse temperature parameter to account for choice randomness. Younger and older adults did not differ in terms of model fits (*t*(1,42) = −0.65, *p* = 0.52). Moreover, simulated choice behavior based on the winning model matched closely the empirical choice behavior observed in both age groups (supplementary Fig. 1).

### 3.2 Age differences in the representation of task-inherent uncertainties

Individual parameter estimates allowed us to assess whether younger and older adults' differ in their task representations, specifically in their estimates of the task-inherent uncertainties (reversal probability and outcome predictability). Fig. 2 shows that younger and older adults did not differ in overall choice accuracy (calculated as percentage of trials when the seasonally appropriate item was chosen). Likewise, estimates of outcome predictability were comparable in both age groups and similar to actual outcome predictabilities (actual outcome predictability 87%, mean estimated outcome predictability in younger as well as older adults 85%, no age group difference (*t*(1,42) = −0.63, *p* = 0.53). However, older adults systematically overestimated the reversal probability of seasons (6% actual reversal probability, mean estimated reversal probability in older adults 13%, in younger adults 9%, age group difference: *z* = −2.72, *p* < 0.01). Computational modeling hence suggests that older adults might perceive states (seasons) as more volatile than they actually are. This means that older adults find it harder to converge on an unequivocal representation of the current state and are more prone to alter their belief about the current season. In line with this, we observed that older adults switched their choices more frequently between winter and summer items, especially if they encountered a loss in the previous trial (supplementary Fig. 3 interaction age group × switching after losses or gains, *F*(1,42) = 7.54, *p* < 0.01, *rICC* = 0.39).

### 3.3 Age differences in updating beliefs about the current season during reversal learning

While model estimates at the individual level allow one to characterize interindividual differences in uncertainties of task representations, model estimates at the trial level enable us to understand what these mean for the development of task representations during learning. We examined trialwise empirical choice behavior and related model estimates after reversals in task states (Fig. 3). As seen in Fig. 2, overall accuracy (mean of correct seasonal choices across all trials) did not differ between age groups (*t*(1, 42) = 0.96, *p* = 0.34). However, when examining age differences in trialwise accuracy after reversals (Fig. 3A), we found that older adults display more accurate behavior shortly after a reversal, but failed to attain levels of choice accuracy as seen in younger adults later after a reversal (*F*(1, 42) = 4.22, *p* < 0.05, *rICC* = 0.30, [interaction age group × trials 1–6 vs. 7–12 following a reversal on choice accuracy]). This speaks to the fact that older adults converge less on an unambiguous representation of the current season, which facilitates the detection of reversals but also prevents them from displaying consistent behavior later on. In a reversal learning task, this is in particular detrimental for choice accuracy later on after reversals, where it manifests as more frequent choices of the wrong option. However, it can result in more frequent (possibly accidentally) correct choices early on after reversals. In line with this, we observed that overall mean accuracy was rather captured in outcome predictability estimates (younger adults *r*(1,25) = 0.34, *p* = 0.09, older adults *r*(1,19) = 0.70, *p* < 0.01), and not in reversal probability estimates (younger adults *r*(1,24) = −0.30, *p* = 0.14, older adults *r*(1,19) = 0.005, *p* = 0.98).

Note that computational modeling suggests that older adults' inconsistent choice behavior is not because of age differences in choice randomness, as individually estimated values for the inverse temperature did not differ between age groups (Fig. 2). Instead, modeling choice behavior suggests that older adults' choices are less consistent because they overestimate reversal probabilities of task states and are consequently less able to converge on a consistent belief about the current season. To further probe the effects of overestimating reversal probabilities on learning in older adults, we examined trial-by-trial estimates of uncertainty about the current season, which was treated as a hidden state that had to be inferred. Uncertainty was defined as the entropy of beliefs about the current season (see Fig. 3B, calculated as $\sum_{i}\log\;P\left(season_{i}\right)$), which is maximal if agents assign uniform probability to both winter and summer (cf. supplementary Fig. 2 and Figs. 3B and C).

Overall, older adults displayed higher uncertainty about the current season (Fig. 3B), again indicative of an impaired ability to converge on an unequivocal task representation. This is particularly prevalent in later trials after a reversal (*F*(1,42) = 4.71, *p* < 0.05, *rICC* = 0.32), where younger adults show stronger evidence in favor of a task representation that reflects 1 particular season because of evidence accumulated over time. As seen in simulations of altered reversal probabilities or outcome predictabilities (supplementary Fig. 4), this effect can be attributed to overestimating reversal probabilities such that the higher the assumed reversal probability, the more difficult it is to reduce uncertainties especially later on after reversals. This is also evident when inspecting beliefs after reversals (Fig. 3C, belief estimate of the currently correct task state, e.g., belief to be in summer state if it is summer). Older adults' belief estimates are overall closer to a uniform distribution and plateau at a lower level when beliefs should be more consistent (Fig. 3C, interaction age group × belief on first half vs. second half of trials after reversals: *F*(1,42) = 13.50, *p* < 0.01, *rICC* = 0.49).

Effects of overestimating reversal probabilities in older adults are also evident in feedback evaluation during learning. Surprise (Fig. 3D) indicates how unexpected a feedback is given a current belief, whereas model updating (Fig. 3E) indicates how much beliefs are changed after an outcome. For older adults, unexpected feedback early after reversals seems less surprising (Fig. 3D, *F*(1,42) = 7.33, *p* < 0.05, *rICC* = 0.39, interaction age group × surprise on trials 1–6 vs. 7–12 after a reversal). This speaks to the fact that older adults are worse at evaluating the unexpectedness of outcomes (especially when they are most unexpected), induced by a higher overall uncertainty about the current season. In addition, later after a reversal, older adults show a trend toward greater model updating as compared with younger adults (Fig. 3E, main effect age group for model updating trials 7–12 after reversals *t*(1,42) = −1.76, *p* = 0.09, no reliable interaction age group × trials after reversal). Older adults thus seem to modify task state beliefs more than younger adults at times when beliefs should be more consistent due to learning.

To summarize, a trialwise analysis of model estimates shows how model-based inference in reversal learning in older adults is affected by increased estimates of the volatility of the current season (reversal probabilities). During choices, older adults are more uncertain whether their current belief about the season is correct. During feedback processing, older adults are more willing to alter their beliefs about the current season. Of note, these effects are particularly pronounced later in time after reversals, when beliefs should be more consistent given accumulated evidence in favor of 1 season. Overestimating reversal probabilities thus manifests as an increased uncertainty in task representations and increased readiness to alter beliefs in older adults (see also supplementary Fig. 4 for a simulation of model-based reversal learning under increased reversal probabilities).

### 3.4 Pupillometric assessment of age differences in belief updating

Computational modeling suggests that older adults are more uncertain in their beliefs about the current state (season) and tend to update their established beliefs more. This suggests older adults are less able to incorporate beliefs about task states into the evaluation of action outcomes. As a validation of our modeling results, we examined age groups differences in the extent to which pupil diameters covary with the relevance of feedback for updating beliefs. As outlined previously, increases in pupil diameter indicate a greater subjective saliency of events, given inferred hidden states. Larger pupil diameters should thus be observed on trials that are more relevant for altering beliefs about the current state. Hence, a difference in the extent to which pupil diameters covary with model updating between age groups can be used to index a difference in the degree to which subjects incorporate task representations when evaluating feedback.

**Fig. 4:**
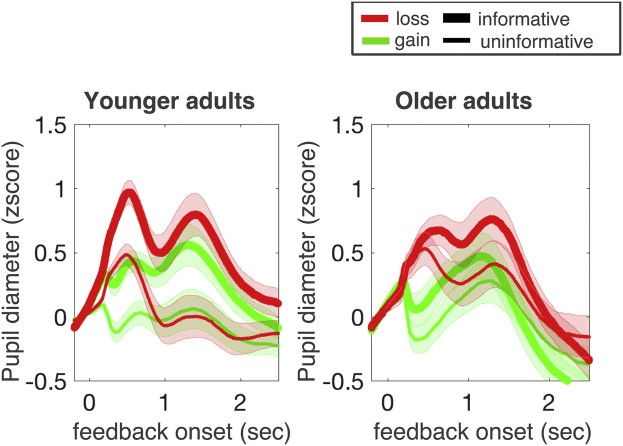
Changes in pupil diameter to feedback on informative trials (choice of seasonal items) and uninformative trials (choice of items not seasonally specific). Older adults differentiate informative and uninformative feedback less, suggesting that they engage less in model-based updating than younger adults. Changes in pupil diameter are z-scored within individuals; lines indicate means within age groups. Shaded areas indicate SEs of mean individual pupil diameters within age groups. Early peaks before 1 second are not included in statistical analyses and reflected lateral positions of gain and loss feedback on the screen.

We first examined whether participants differentiated between outcomes that did or did not allow them to learn about the current season, by examining mean pupil responses during feedback for informative and uninformative trials. As outlined previously, uninformative trials did not allow subjects to learn about the current season and should therefore elicit smaller pupil dilations as compared with informative trials. Note feedback on informative trials was physically identical to feedback on uninformative trials and similarly frequent in valence and expectedness. As predicted, we found larger pupil diameters during feedback that was informative for learning about task representations (mean feedback response on informative trials, cf. Fig. 1) compared with the feedback response on uninformative trials (cf. Fig. 4A). However, only in younger adults were there stronger responses for informative feedback (*F*(1,24) = 39.11, *p* < 0.05, *rICC* = 0.80, main effect informativeness in younger adults). Older adults instead reacted equally to feedback that was informative and feedback that was uninformative (*F*(1,19) = 2.49, *p* = 0.14; no main effect informativeness in older adults, *F*(1, 42) = 9.01, *p* < 0.05, *rICC* = 0.42, age group × informativeness interaction). Post hoc power analysis showed that our sample size allowed us to detect difference effects of at least d = 0.60 and larger with a power of at least 80% in older adults. The observed effect size in younger adults was d = 1.2. Older adults thus seemed less able to suppress the subjective salience of feedback which was uninformative for updating task representations.

In addition, across age groups, we observed a stronger response to loss as compared with gain feedback (*F*(1, 42) = 8.68, *p* < 0.05, *rICC* = 0.41), especially on informative trials (interaction loss—gain × informative—uninformative trials *F*(1, 42) = 9.01, *p* < 0.05, *rICC* = 0.42). Negative events thus appear more salient, independent of the expectedness of negative events (as expectedness, informativeness and frequency of gains and losses was balanced).

**Fig. 5:**
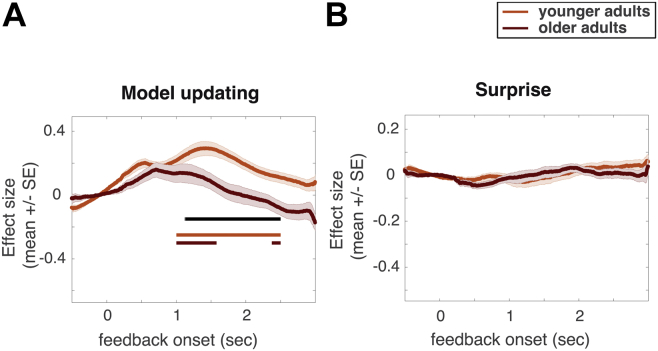
Regression results on trialwise changes in pupil diameter. (A) In both age groups, positive betas of the trialwise pupil prediction show that pupil diameter is larger on trials with more model updating (red and orange lines indicate time points of a reliable [*p* < 0.05] positive effect for older and younger adults, respectively). This effect is stronger in younger adults (time points of a reliable age differences indicated by black line). (B) Trialwise changes in surprise were not related to changes in pupil diameter. Regression lines indicate means of betas across individual regressions within age groups; shaded areas indicate SEs across individuals within age groups. Regressions calculate betas per sample point, time points of reliable effects are determined based on permutation tests in time window 1–2.5 seconds after feedback onset. (For interpretation of the references to color in this figure legend, the reader is referred to the Web version of this article.)

In a second set of analyses, we examined whether trialwise changes in pupil dilation covaried with model updating on informative trials. A positive link between pupil dilation and the amount of model updating would suggest an increased salience of feedback that was more relevant for learning. Based on a reduced differentiation of informative and uninformative feedback in older adults, we would expect that they also differentiate trials of large or small model updating less as compared with younger adults. To examine trialwise relationships between model estimates and pupil diameters, we used a regression which predicted pupil diameter on a given trial. Regressions used a trialwise estimate of model updating (cf. Fig. 3E, supplementary Fig. 2C), and surprise (cf. Fig. 3D, supplementary Fig. 2D), as well as regressors of no interest (feedback valence, changing seasonal ratings) to predict pupil diameters. As seen in Fig. 5A, pupil diameters were positively related to model updating in both age groups, with larger pupil diameters seen on trials with more model updating. Interestingly, this effect was weaker in older adults (time window of significant age difference in the size of the updating effect indicated by black line in Fig. 5A, *t*(1,42) = 3.55, *p* < 0.05). Inspecting pupil dilation as an indicator of subjective salience thus suggests that older adults pay greater attention to outcomes that do not provide for learning about task states (uninformative trials) and modulate pupil dilation less with trials that inform a change in model updating.

Finally, orthogonalizing informativeness and expectedness of outcomes in our task allowed us to address whether pupil responses differentiate the mere unexpectedness of an event (surprise) from its informativeness for learning about task representations (model updating). Unlike model updating, trialwise differences in surprise (unexpected outcomes without changes in beliefs) were not reliably reflected in changes in pupil diameter (Fig. 5B) in either age group. This clear separation of model updating and surprise in pupil diameters is not unprecedented (Nassar et al., 2012, O’Reilly et al., 2013) but adds to an emerging picture regarding the precise sensitivity of an arousal system. In the context of the present study, we find that surprising events are not as subjectively salient as events that allow for model updating. This might be due to the fact that participants were instructed to learn about the current season and hence focused on model updating. However, at least in the case of the older adults, a reduced response to surprising events is unlikely to be attributed to disengaging from the task as they also showed increased pupil diameters on uninformative trials.

To summarize, at an interindividual level, we observe older adults overestimate the changeability of task states and commit less to a given task state belief. This means that, compared with younger adults, older adults are consistently less certain in their belief about the current season and are less able to converge on an unequivocal task representation through learning. In line with this, age differences in pupil dilations during feedback processing suggest that older adults are less able to focus on feedback that is informative for learning about task representations. Instead, uninformative feedback appears to be similarly relevant for them and they differentiate less between feedback that allows for more or less model updating.

### 3.5 Trialwise ratings on task states

**Fig. 6:**
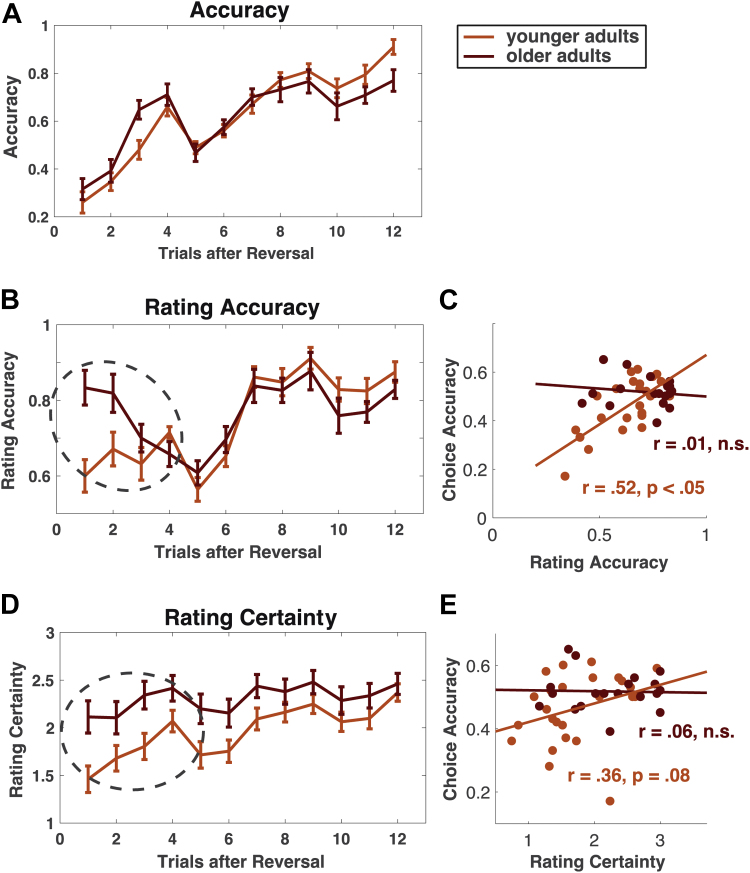
Rating data for currently assumed task states (B) and certainty about task state classification (D). Older adults are less able to capture their current choice accuracy (A) in ratings about the current task state (C and E).

Finally, as an additional measure of individual task representations in both age groups, we asked participants at the end of every trial whether they thought the current season was winter or summer and how certain they were in this assessment (on a scale from 1 to 4, see Fig. 1 and Methods). Interestingly, this revealed that older adults' accuracy and certainty in their ratings were less in line with their actual choice performance than was the case for younger adults. As shown previously, both age groups display lower choice accuracy immediately after reversals (Fig. 6A). However, while younger adults showed slowly increasing rating accuracy and rating certainty after reversals, older adults showed higher levels of rating accuracy and certainty already early on after reversals (Fig. 6B, interaction age group × rating accuracy on first half vs. second half of trials after reversals: *F*(1,42) = 7.02, *p* < 0.05, *rICC* = 0.38; Fig. 6D, interaction age group × rating certainty on first half vs. second half of trials after reversals: *F*(1,42) = 5.60, *p* < 0.05, *rICC* = 0.34). Indeed, older adults' overall indicated certainty in their beliefs about the current season was higher than in younger adults (*F*(1,42) = 4.70, *p* < 0.05, *rICC* = 0.32), although their choice behavior suggested that they are less certain as compared with younger adults (see also higher choice entropy in older adults in Fig. 3D). The correlation between choice accuracy and rating certainty or rating acccuracy in the first half after reversals (trials 1–6) was therefore overall weaker in older adults than in younger adults (Fig. 6C and E, correlation choice accuracy and rating accuracy: *r* = 0.52, *p* < 0.05, 95% CI 0.49 to 0.65 in younger adults; *r* < 0.01, *p* = 0.99, 95% CI −0.12 to 0.13 in older adults; correlation choice accuracy and rating certainty: *r* = 0.36, *p* = 0.07, 95% CI 0.30 to 0.49 in younger adults; *r* = 0.06, *p* = 0.79, 95% CI −0.03 to 0.21 in older adults). This suggests that older adults track their actual choice accuracy less in their ratings of task state uncertainty. It should be noted that this does not reflect a general inability of older adults to differentiate task state certainty, as both younger and older adults indicated lower certainty about the current task state on uninformative as compared with informative trials (mean certainty [on a scale from 0 to 3] on uninformative trials 1.38 and 1.46 for younger and older adults, respectively, and 2.03 and 2.30 on informative trials). Instead, this provides further evidence that older adults are more disposed to switch task state assumptions and hints at a metacognitive deficit in older adults in evaluating current choice certainty precisely in conditions of updating task representations.

## 4 Discussion

We investigated whether younger and older adults differ in their ability to form adequate task representations in decision-making using a probabilistic reversal learning paradigm, and availing of computational modeling and pupillometry. The computational model based on a Bayesian learner captured the inherent uncertainties of the task paradigm (estimates of reversal probability and outcome predictability) and allowed us to compare younger and older adults in their ability to form adequate representations of uncertainties in decision-making. The data suggest that inconsistent choice behavior in older adults is attributable to overestimating the reversal probability of task states. This means older adults are less able to converge on an unequivocal and sustained task representation (is it winter or summer), even if evidence that favors 1 task state is available.

### 4.1 Consequences of overestimating reversal probabilities in older adults

At a trial-by-trial level, overestimating reversal probabilities manifests as higher levels of choice uncertainty (belief entropy), which cannot be reduced through learning, as well as a greater tendency to alter established beliefs about task states. Choice data suggest that this increased readiness to switch beliefs about the current season in older adults was strongly guided by negative outcomes. Importantly, our task design enabled us to decouple the valence of outcomes from outcome informativeness (for updating task representations) or outcome expectedness. Our analysis shows that older adults' choice behavior is more driven by superficial features of action outcomes as compared with their informativeness with respect to their internal representations. A greater tendency in older adults to be guided by external cues has been observed in many domains of higher cognitive functioning (Lindenberger and Mayr, 2014). Here we show in model-based reversal learning this can be understood as reflecting a reduced ability to form precise internal representations of optimal choice behavior.

The fact that older adults ratings of choice certainty and choice accuracies were less in line with their actual choice behavior, compared with younger adults, can be seen as further confirmation of their inability to form or access precise internal representations of task contingencies. Instead, older adults generally overestimated the certainty of their current task state classification and seemed less conflicted in task state ratings especially immediately after reversals, as compared with younger adults. Interestingly, a similar metacognitive deficit in older adults is observed in the memory domain. Here, high certainty ratings for inaccurate responses are observed in the form of high confidence memory errors (Shing et al., 2009). However, unlike memory studies, reinforcement learning studies which use computational models allow for a precise estimation of the possible confidence in the current response and provide an interesting new avenue for further investigating metacognitive deficits in older adults.

### 4.2 Specific deficit of evaluating outcomes based on internal task representations in older adults

In addition to computational estimates of task representations and metacognitive ratings, our study also assessed changes in pupil diameter. Increased pupil diameter can be indicative of increased noradrenergic modulation and is linked to greater subjective relevance of events. Indeed, we observed larger pupil diameters for informative outcomes and in particular those that allowed to update beliefs about task states in younger adults. However, in line with a reduced ability of older adults to assess the relevance of events based on internal task representations, we found that older adults responded strongly to feedback that allowed for updating beliefs about task states, as well as to feedback that did not allow for updating of beliefs. In addition, pupil diameters in older adults varied less with how much learning about task states was possible, if outcomes allowed for updating beliefs. This suggests that older adults indeed show a specific deficit in evaluating outcomes based on internal task representations. This altered outcome processing in older adults cannot be attributed to a disengagement from the task as mean accuracy levels, as well as ratings on task commitment, were comparable between younger and older adults. Likewise, pupil responses were not generally reduced in older adults.

Computational modeling as well as pupillometric results instead point to an altered relevance of task representations during model-based reversal learning in older adults. The precision of task representations and the impact of model updating at outcome presentation depend on each other as learning unfolds. A common problem in understanding age differences in model-based reversal learning is to disentangle whether older adults' ability to process outcomes is impoverished because they are less able to develop precise task representations, or whether they struggle with a reduced ability to develop task representations because their ability to process feedback gainfully is reduced (a favored hypothesis in many studies (de Boer et al., 2017, Hämmerer and Eppinger, 2012). Our study is a step toward disentangling these 2 explanations. We included a condition that assessed feedback evaluation when it could not be used for learning about task state beliefs (uninformative trials), thereby breaking the link between feedback response and updating beliefs about task representations. In this condition, we observed notable age-group differences consistent with older adults taking task representations less into account when judging feedback. It is interesting to speculate that this age effect may be in part a reaction to a reduced ability to form reliable internal task representations because of, for example, increased neuronal noise in older adults (Allen et al., 1998, Li et al., 2001). Interestingly, our results showed that older adults rather react too strongly to uninformative events, which refutes the notion that older adults struggle to learn from outcomes as they cannot respond physiologically to the degree that younger adults do (Eppinger et al., 2013a, Hämmerer and Eppinger, 2012). Instead, it suggests an inability to form precise expectations toward action outcomes has to be at least also taken into account when comparing age differences in learning from feedback and reacting to feedback physiologically.

Unlike prior studies on age differences in reinforcement learning, our study assessed age differences in task representations independent of age differences in the expectation of gains or losses. Age differences in assessing the relevance of gains and losses have been reported previously (Eppinger et al., 2013a, Hämmerer et al., 2011, Larkin et al., 2007) and might affect expectations toward outcomes if these are not controlled for. Our paradigm kept gains and losses independent from performance accuracy, such that age groups did not differ in expected frequencies of gains in our task, even in the presence of performance differences. This enabled us to disentangle the expectancy of receiving a gain from the precision of task representations (i.e., the certainty with which 1 can expect a gain [or any] outcome) and allowed greater control in examining age differences in task representations on the 1 hand and the impact of positive or negative outcomes on the other hand. We observed that both age groups showed stronger reaction to loss as compared with gain outcomes, independent of the informativeness of expectedness of outcomes. This confirms earlier suggestions that negative outcomes indeed carry more subjective salience in feedback-based learning task (Hämmerer and Eppinger, 2012).

### 4.3 Modulation of pupil diameters with model updating, but not surprise

By orthogonalizing expectedness and informativeness of outcomes, our novel reversal learning paradigm enabled us further to address the relevance of model updating and surprise in feedback processing. As a general principle, greater model updating can be assumed during outcomes that are less expected but informative for learning about task states. By contrast, more surprise can be assumed during outcomes that are less expected but uninformative. In both age groups, we observed larger pupil diameters with more model updating, but no modulation of pupil diameters with different degrees of surprise. Our study contributes to an unfolding picture of the selectivity of pupil dilation (and possibly noradrenergic modulation), which however yielded inconsistent results, with one study showing pupil dilation to reflect model updating (Nassar et al., 2012) and another study finding that pupil dilation is stronger for surprising events as compared to model updating (O'Reilly et al., 2013). To understand these disparate results, a more general approach to interpreting pupil diameters is needed which identifies relevant events task by task. For instance, an unexpected event that allows for model updating can be more relevant in a task where performance ultimately depends on adjusting behavior quickly (Nassar et al., 2012, and this study). By contrast, in other tasks, an unexpected event which does not allow for learning, but necessitates a specific response (as, e.g., in oddball tasks), might be considered more relevant (Aston-Jones et al., 1994, O’Reilly et al., 2013).

Finally, we note that postmortem evidence on neuronal loss in the noradrenergic LC during aging suggests a reduced potential for noradrenergic modulation in older adults (Mann, 1983, Mather et al., 2015). In the context of this study, 1 might expect to see overall reduced pupil dilation, reflecting an overall reduced LC functionality in older adults. Indeed, a recent study observed smaller pupil dilation to salient negative events in older adults (Hämmerer et al., 2018). In the present study, we did not find lower pupil dilation in older adults. However, this is not conclusive evidence against reduced noradrenergic levels in older adults. As outlined previously, if an event is salient given its relevance in a particular task context, the strength of a functional response will also depend on a given task focus. In addition to age differences in biological substrates for processing salient events, age differences in attentional focus on salient events need to be taken into account. As processing salient events (outcomes) in our task happened within a task context that required a focus on outcomes, we cannot address these 2 aspects separately in this study. Indeed, pupillometric responses of comparable strength in younger and older adults are not unprecedented in feedback-based learning tasks (Hämmerer et al., 2017). In the context of our task, this suggests that the strength of attentional focus on outcome evaluation might have been comparable in younger and older adults. However, adaptation to evaluating outcomes in light of current task state representations was reduced in older adults, evident in a reduced differentiation of outcomes that allowed learning about task state representations.

## 5 Conclusion

To conclude, we show that older adults form less reliable beliefs about task states and have more difficulties in updating task representations through learning from choice outcomes. This reduced ability to converge on unequivocal internal task representations in older adults was accompanied by an inaccurate description of choice accuracies and choice certainties in confidence ratings, an increased tendency to guide choices by external cues as well as a reduced ability to evaluate outcomes based on internal task representations. A deficit in forming or maintaining precise task representations has been proposed to underlie cognitive deficits in several cognitive domains during aging. Our study sheds new light on the consequences of inadequate task representations during model-based reversal learning in older adults.

## Disclosure

The authors have no financial or nonfinancial competing interests.
