# Supplementary material for: Older adults fail to form stable task representations during model-based reversal inference
Source: Neurobiol Aging. 2019 Feb;74:90–100. doi: 10.1016/j.neurobiolaging.2018.10.009 (PMC6338680; doi:10.1016/j.neurobiolaging.2018.10.009)
Supplement: Supplemental Material [file mmc1.docx]

**Supplementary material**

Supplementary Methods

*Computational modelling*

We performed model comparison between Bayesian belief updating (Hidden Markov Model, HMM), three variants of Rescorla-Wagner learning (Rescorla and Wagner, 1972) and a simpler ‘switching’ model of this task (see supplementary Methods and supplementary Table 1). The details of these models have been described in detail previously (FitzGerald et al., 2017; Schwartenbeck et al., 2016)).

The Bayesian or Hidden Markov Model casts the task to find the true season (context) as an inference problem (model A), where the current season is treated as a latent variable $x_{t}$ at time-step $t$ with two possible states (winter or summer). Agents are assumed to perform inference on the current season based on a generative model with the form:

$P\left( x_{t} | o_{t},y_{t},x_{t-1},A,B \right)=\frac{P(o_{t}|x_{t},y_{t},A)\cdot P(x_{t}|x_{t-1},B)}{P(o_{t})}$ (1)

Here, $o_{t}$ refers to the feedback ($positive=1$ or $negative=2$) and $y_{t}$ refers to the received item (summer or winter item, or both). Note that, given that sometimes the unchosen item was delivered in our task, subjects had to perform inference based on the item they received rather than the item they chose.

The likelihood function $P\left( o_{t} | x_{t},y_{t},A \right)$ was defined with respect to an observation function of the form:

$$P\left( o_{t}=1 | x_{t}=i,y_{t}=j,A \right)=A_{1ij}$$

$$A_{1**}=\left[ \begin{matrix} \nu& 1-\nu\\ 1-\nu& \nu\end{matrix} \right]$$

(2)

$$P\left( o_{t}=2 | x_{t}=i,y_{t}=j,A \right)=A_{2ij}$$

$$A_{2**}=\left[ \begin{matrix} 1-\nu& \nu\\ \nu& 1-\nu\end{matrix} \right]$$

The parameter $\nu$ reflected *outcome predictability* (i.e., cue validity) and was treated as a free parameter that was estimated given observed behavior.

The prior over the current season $P(x_{t}|x_{t-1},B)$ was defined as the posterior belief from the previous trial modulated by the reversal probability $\rho$ (i.e., the transition probabilities between hidden states or seasons):

$B=\left[ \begin{matrix} \rho& 1-\rho\\ 1-\rho& \rho\end{matrix} \right]$ (3)

Here, $1-\rho$ encodes the probability of a reversal between seasons from one trial to the next, added per trial. We treated $\rho$ as a second free parameter that was fitted to individual behavior.

Note that $P\left( o_{t} \right)=\sum_{i} P\left( o_{t} | x_{i,t},y_{t},A \right)\cdot P(x_{i,t}|x_{t-1},B)$ with $i\in\{winter,summer\}$.

Finally, choice probabilities for different actions $a_{t}$ (i.e, choosing option 1 and option 2) were determined by using a softmax decision rule based on an agent’s current prior beliefs about the season and an inverse temperature parameter *γ*:

$P\left( a_{t},\gamma\right)=\frac{e^{\gamma\cdot P(x_{t}|x_{t-1},B)}}{\sum_{i} e^{\gamma\cdot P(x_{i,t}|x_{t-1},B)}}$ (3)

We compared this HMM to different variants of Rescorla-Wagner (RW) learning in this task (FitzGerald et al., 2017). First, we used a standard RW-learning model (model B), where the value of selecting the winter or summer item is updated based on positive or negative feedback in a trial $t$:

$V_{chosen item,t+1}=V_{chosen item,t}+ \alpha\left( R_{t}-V_{chosen item,t} \right)$ (4)

V indicates the value of the chosen Stimulus item, α reflects the learning rate, which was treated as a free parameter, and R indicates the reward obtained on that trial t. Choices were again determined based on a softmax decision rule with an inverse temperature $\gamma$

$P(choice)= \frac{e^{\gamma V}}{\sum_{i} e^{\gamma V_{i}}}$ (5)

The values of the winter and summer items were initialized to 0.5.

Given that participants can be expected to simultaneously learn about the value of the unchosen option as well, we also examined a RW-learning model (model C), which also updated the value of the unchosen option, such that:

$V_{unchosen item,t+1}=1-V_{chosen item,t+1}$ (6)

This model was otherwise identical to model (B).

Further, we tested a RW-model (D) that included a trial-by-trial adjusted learning rate depending on the size of the prediction error (Diederen and Schultz, 2015), because learning rates can be expected to be larger immediately after reversals. The values of the items were determined as in Model (C), but the trial-specific learning rate was defined as:

$\alpha_{(t)}=\mu* \left| \left( R_{t}-V_{chosen item,t} \right) \right|+(1-\mu)*\alpha_{t-1}$ (7)

Here, µ indicates the weighing factor that regulates the change of the learning rate depending on the absolute size of the prediction error. Consequently, this model had three free parameters (α_,_ $\gamma$ and µ).

Finally, subjects could also apply simpler, less ‘model-based’ response strategies (Summerfield et al., 2011). We therefore also tested a simple stay-switch model (E), where the (deterministic) beliefs about the current season were fully determined by the feedback of the previous trial. If winter items yielded a gain (whether chosen or not) on the previous trial, agents would have a preference for chosing winter on the following trial, and the same for summer. This model only had the inverse temperature $\gamma$ in the softmax decision rule as a free parameter.

*Model fitting and model comparison*

To obtain individual parameter estimates, maximum a posteriori (MAP) estimation was performed using moderately informed priors based on the true parameter values to regularize the maximum-likelihood solution (reversal probability $\rho$: Beta(47.5,2.5), reliability of feedback $\nu$: Beta(47.5,2.5), learning rate α for the RW-learning models: Beta(25,25), and inverse temperature ln(γ): N(2,0.5)). Model inversion was carried out using the Nelder-Mead method, and random effects Bayesian model comparison (Rigoux et al., 2014; Stephan et al., 2009) was performed based on the Bayesian Information Criterion (BIC) of each model. All trials were included in the model inversion of the Bayesian belief updating and RW-learning models to obtain individual parameters, perform model comparison and simulate choice performance as described below.

| ***Younger Adults (n = 25)*** | | | | | |
| --- | --- | --- | --- | --- | --- |
| **Modelname** | **Free parameters** | **Summed model evidence (current-best model)** | **Summed model probability (alpha)** | **Posterior probability** | **Model fit (r^2^)** |
| **Model A**  HMM | 3 (Reversal probability, feedback reliability, inverse temperature) | 0 | .87 | 1 | .42 |
| **Model B**  RW learning | 2 (Learning rate, inverse temperature) | -412 | .03 | <.01 | .29 |
| **Model C**  RW learning with simultaneous updates | 2 (Learning rate, inverse temperature) | -151 | .03 | <.01 | .40 |
| **Model D**  RW-learning with adaptive learning rate | 3 (Learning rate, inverse temperature, weight for learning rate) | -504 | .03 | <.01 | .30 |
| **Model E**  Stay-switch model | 1 (Inverse temperature) | -513 | .03 | <.01 | .32 |

| ***Older Adults (n = 19)*** | | | | | |
| --- | --- | --- | --- | --- | --- |
| **Modelname** | **Free parameters** | **Summed model evidence**  **(current-best model)** | **Summed model probability (alpha)** | **Posterior probability** | **Model fit (r^2^)** |
| **Model A**  HMM | 3 (Reversal probability, feedback reliability, inverse temperature) | 0 | .82 | 1 | .37 |
| **Model B**  RW learning | 2 (Learning rate, inverse temperature) | -427 | .04 | <.01 | .18 |
| **Model C**  RW learning with simultaneous updates | 2 (Learning rate, inverse temperature) | -181 | .04 | <.01 | .31 |
| **Model D**  RW-learning with adaptive learning rate | 3 (Learning rate, inverse temperature, weight for learning rate) | -493 | .04 | <.01 | .19 |
| **Model E**  Stay-switch model | 1 (Inverse temperature) | -227 | .05 | <.01 | .32 |

Supplementary Table 1. Overview model fits within age-groups

Supplementary Table 2

|  | Younger Adults | | Older Adults | |
| --- | --- | --- | --- | --- |
|  | Informative | Uninformative | Informative | Uninformative |
| Gain valid | 47 (0.005) | 43 (0.001) | 47 (0.008) | 44 (0.003) |
| Loss valid | 39 (0.005) | 45 (0.001) | 40 (0.008) | 45 (0.003) |
| Gain overall | 54 (0.003) | 50 (0.001) | 55 (0.007) | 50 (0.004) |

Supplementary Table 2. Frequency of gains and losses on informative and uninformative as well as expected and unexpected (probabilistic). Shown are feedback types in percent for informative and informative trials (standard error in brackets).

Supplementary Figure 1

Supplementary Figure 1. Model fits of the Bayesian belief-updating model after season reversals. Simulated choice behavior based on Bayesian belief-updating (winning model) matched closely the empirical choice behavior observed in both age-groups.

Supplementary Figure 2

Supplementary Figure 2. Example Participant, overview of relationship between model estimates across trials. Depicted are trials as sorted by ascending belief (belief estimates included as blue line) of the current season in place (summer belief given summer task state, or winter belief given winter task state). Beliefs below 0.5 are thus incorrect assumptions given the current task state). A and B) Belief uncertainty and response uncertainty are maximal when beliefs (blue line) are around 0.5 (dotted black line) and thus least in favour of one particular season. Response uncertainty in B) is 0 for uninformative trials. C) and D) Model updating as well as surprise is larger when beliefs favour the wrong task state assumption (beliefs < 0.5), these are also often (informative) trials immediately following task state reversals (green circles indicate feedback on the first informative trial after reversals) or probabilistic feedback (black circles). D) Unlike model updating, stronger surprise can also be found for probabilistic feedback on uninformative trials (black circles in (C) with model updating = 0 and in (D) with suprise > 1.5).

Supplementary Figure 3

Supplementary Figure 3. Switching between chosing winter and summer items after gains or losses. Older adults switch more frequently after losses between seasons. Data are shown across informative and uninformative trials, percentages are % of switching between seasons versus staying with previous seasonal choice for previous gain or loss trials, respectively. Bars indicate means per age groups, errorbars SEs, asterisks indicate reliable group differences at p <.05.

Supplementary Figure 4

Supplementary Figure 4. Impact of altered reversal probability or cue validity estimates on trial-wise estimates of beilef entropy, belief, model updating and surprise after reversals. Orange and red lines indicate trialwise parameter estimates based on actual choice behavior in younger and older adults, respectively (cf Figure 3). Black dotted lines show simulated trialwise parameter estimates based on an ideal capture of task parameters in choice behavior (reversal probability of .95 and outcome predictability of .85). Blue dotted lines indicate variations in simulated trialwise parameters estimates with different reversal probabilities and outcome predictability = .85. Green dotted lines indicate variations in simulated trialwise parameters estimates with different outcome predictabilities and reversal probability = .95. Age differences in trialwise estimates of belief entropy, belief, model updating and surprise are best captured by age differences in reversal probabilities (left columns). It is evident that overestimating reversal probabilities (as is the case in older adults) reflects most prominently in an inability to lower belief entropies even lateron after reversals (first row). In contrast, altered estimates of outcome predictability have strongest consequences on model updating (third row). The lower the assumed outcome predictability, the lower and the less differentiated the trialwise estimates of model updating.
